# Quality of life among people living with hypertension in a rural Vietnam community

**DOI:** 10.1186/1471-2458-14-833

**Published:** 2014-08-11

**Authors:** Ninh Thi Ha, Hoa Thi Duy, Ninh Hoang Le, Vishnu Khanal, Rachael Moorin

**Affiliations:** Institute of Public Health, Ho Chi Minh City, Vietnam; School of Public Health, Curtin University, Perth, Western Australia Australia; Faculty of Health Science, Curtin University, Perth, Western Australia Australia

**Keywords:** Hypertension, Quality of life, Rural area, WHOQOL-BREF, Vietnam

## Abstract

**Background:**

To respond to growing prevalence of hypertension in Vietnam, it is critical to have an in-depth understanding about quality of life (QOL) among people living with hypertension and related factors. This study aimed to measure QOL among hypertensive people in a rural community in Vietnam, and its association with socio-demographic characteristics and factors related to treatment.

**Methods:**

This study was conducted in a rural community located 60 km from Ho Chi Minh City. Face-to-face interviews were conducted among 275 hypertensive people aged 50 years and above using WHOQOL-BREF questionnaire. Descriptive statistics were used to examine mean scores of quality of life. Cronbach’s alpha coefficient and Pearson’s correlation coefficient were applied to estimate the internal consistency, and the level of agreement between different domains of WHOQOL-BREF, respectively. Independent *T*-test and ANOVA test followed by multiple linear regression analyses were used to measure the association between QOL domains and independent variables.

**Results:**

Both overall WHOQOL-BREF and each domain had a good internal consistency, ranging from 0.65 to 0.88. The QOL among hypertensive patients was found moderate in all domains, except for psychological domain that was fairly low (mean = 49.4). Backward multiple linear regressions revealed that being men, married, attainment of higher education, having physical activities at moderate level, and adherence to treatment were positively associated with QOL. However, older age and presence of co-morbidity were negatively associated with QOL.

**Conclusion:**

WHOQOL-BREF is a reliable instrument to measure QOL among hypertensive patients. The results revealed low QOL in psychological domain and inequality in QOL across socio-demographic characteristics. Given the results, encouraging physical activities and strengthening treatment adherence should be considered to improve QOL of hypertensive people, especially for psychological aspect. Actions to improve QOL among hypertensive patients targeted towards women, lower educated and unmarried patients are needed in the setting.

## Background

Hypertension is the most important risk factor of cardiovascular and kidney disease; and a leading risk factor for mortality [1]. At least 7.1 million people in worldwide die each year as a consequence of hypertension [2]. In 2008, nearly a billion adults aged 25 years and older had hypertension, and three quarters of the number were living in developing countries [3]. However, despite such high prevalence awareness and blood pressure control are fairly poor in developing countries as a result of inadequate access to information, healthcare facilities, inappropriate dietary habits, poverty and high cost of medications [4].

In Vietnam, hypertension has become an important public health problem and one of leading causes of death and morbidity in hospitals [2]. While it accounted for approximately 1% of population in 1960, by 1991 it increased to 11.2% [5]. A recent study estimated that hypertension currently affects 25% of the adult population in Vietnam [2]. However, the level of awareness and efforts to control of hypertension remains relatively low [2, 6]. A recent national survey found that among hypertensive people only a half (48.4%) were aware of their high blood pressure and only a third (29.6%) were undertaking treatment [2]. In addition, only a third of the patients undertaking treatment had their blood pressure controlled [2].

Quality of life (QOL) is an important indicator to evaluate hypertensive treatment outcomes. A recent systematic review of 20 studies indicated that hypertensive patients had a lower QOL compared with normotensive people [7]. The QOL of hypertensive patients tends to be worse among those with co-morbidity [8–10]. In Vietnam, a number of studies have examined QOL among older people; and people living with HIV and AIDS [11–14]. To best of our knowledge, there is no study measuring QOL among hypertensive people in rural area, especially in the southern part of Vietnam. Understanding QOL of individuals living with hypertension will help policy makers and healthcare managers design and implement culture specific support and care. Thus, this study aimed to examine the QOL among people living with hypertension in a rural area in Vietnam in four dimensions (physical health, psychological, social relationship and environment) using the World Health Organization Quality of life - BREF instrument (WHOQOL-BREF) [15] and its association with socio-demographic characteristics and factors related to treatment.

## Methods

### Subjects and study design

This was a cross –sectional study conducted between February and March 2013 in a rural commune of Long An province named Phuoc Loi located about 60 km West of Ho Chi Minh City. The commune is a pilot setting for screening hypertension among people aged 50+ years.

The sample size required for the study was estimated using a formula of estimating the mean [16, 17]. The mean of score of QOL of the studied population was estimated at 5% of type I error and a standard deviation, based on previously unpublished study [18], is 8.1 QOL scores. Using a precision of one QOL score of either side instead of five QOL scores of either side like the previously unpublished study [18] to increase sample size, the required sample size was 252 people. After further accounted for a non-response rate of 10%, the total sample size required was 275 people.

According to a requirement of the National Targeted Program for Management of Hypertension, hypertensive patients are managed and provided treatment at commune health stations. As a pilot setting in screening for hypertension the number of hypertensive patients at Phuoc Loi is updated annually. In January 2013, a total of 389 hypertensive patients were managed at Phuoc Loi commune health station. The list of patients at the commune health station was used as a sampling frame to select participants. A random sampling methodology was employed to select 275 hypertensive people aged 50+ years for interviews.

Eligible participants completed the structured questionnaire by face-to-face interview conducted by trained interviewers at their homes. The interviewers were trained and involved in pre-testing the Vietnamese version of questionnaire.

### Instruments

The WHOQOL-BREF questionnaire developed by World Health Organisation, a short form of WHOQOL-100, is a cross-cultural instrument. The instrument can capture broadly and totally all aspects of QOL including physical health, psychological, social relationship and environment [15]. As WHOQOL-BREF does not impose a great burden on the respondent it is seen as the most useful instrument to assess QOL [15].

The WHOQOL-BREF was employed in the study to measure QOL among hypertensive patients. It contains two items from the Overall QOL and general Health and 24 items of Satisfaction with rating on a 5-point Likert scale [19]. The 24 items were divided into four domains: Physical health with 7 items (DOM1), Psychological health with 6 items (DOM2), Social relationships with 3 items (DOM3) and Environmental health with 8 items (DOM4). Each item of the WHOQOL-BREF is scored from 1 to 5 on a response scale. The English version of WHOQOL-BREF was translated into Vietnamese language and used in an unpublished study [18]. The questionnaire was pretested among 25 local people to adjust wording before data collection.

### Ethics

The ethical approval for the study protocol was obtained from the Scientific Board of the Institute of Hygiene and Public health at Ho Chi Minh City. The study was explained to all participants and written informed consent was obtained prior to conducting the interviews. Participants were informed that they had the right not to participate or withdraw from the study any time; and that any inability to participate would not disadvantage them in their treatment and care.

### Definition of variables

#### Outcome variables

Four domains of WHOQOL-BREF instrument were outcome variables. The raw score of each domain was transformed directly to be comparable with the scores derived from WHOQOL-100. The score of each domain was then re-transformed to a 0–100 score with a higher scores denoting higher QOL. The details of the steps are presented in the WHO guidelines [20].

#### Independent variables

**Figure 1:**
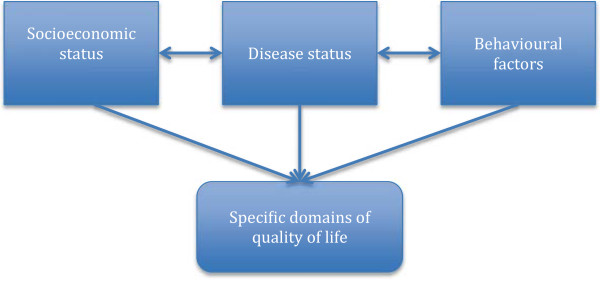
**Conceptual framework of factors associated with quality of life in hypertensive patients.**

Independent variables were selected based on the previously published studies [6, 21] and a conceptual framework adapted from the model described by Wilson and Cleary, and Vidrine and et al. (Figure 1) [22, 23]. Information was collected about age, sex, education (under secondary school and higher), working status (current working and not working), marital status (married and single/widowed/divorced/separated), and religion (no religion, Buddhist and others). The economic status of each household was categorised into normal households and poor households. Poor household were defined as those currently holding a national poor household document provided by the local government (income less than US$20 per capita per month) [24]. Duration of illness was calculated using the date of diagnosis with hypertension to the date of completion of the survey categorised as follows: less than one year, one to less than 5 years, 5 years to less than 10 years and 10+ years. Patients were classified as adhering to treatment if they attended follow-up appointments and took medication appropriately. Self-report of a presence of co-morbidity included diabetes, kidney diseases, cardiac diseases, stroke and arthritis. Physical activities were measured using the International Physical Activity Questionnaire short form, classified into low, moderate and high level [25].

### Statistical analyses

To our knowledge, no study to date has provided evidence of validity and reliability of WHOQOL-BRIEF in Vietnamese version in hypertensive patients. In order to the validity of the instrument for assessment QOL among the hypertensive patients, Cronbach’s alpha was used to estimate the reliability of the WHOQOL-BREF. Based on other studies, Cronbach’s alpha values of 0.70 and over were deemed acceptable [21, 26]. Pearson’s correlation coefficient was used to determine the level of agreement between the four domains of the WHOQOL-BREF. Correlations >0.4 were considered acceptable [21, 27]. The same tests for validating WHOQOL-BREF have been used previously [21].

In the study, descriptive analyses performed including frequencies, percentages, ranges, means, and standard deviations (SD). Independent t- test and ANOVA tests were used to examine the association between participants’ characteristics and average WHOQOL-BREF domain scores.

All the associated factors were further examined by multivariate linear regression using a stepwise backward elimination procedure with cut off point 0.05. Transformed scores were used for statistical analyses in the four domains. In this study, the level of significance was set at *p* < 0.05 for all analyses.

## Results

### Characteristics of the study populations

The participant characteristics are presented in Table 1. The mean age of the 275 participants was 65.8 years (SD 9.9 years), 40.7% were men, 40.0% had secondary school education or above, 36.7% were current working, 45.8% were Buddhist and 4.7% were from poor households. Most participants (73.5%) had been diagnosed with hypertension for more than one year, and more than a half (53.5%) reported compliance with treatment. Cardiac disease (15.3%), arthritis (14.2%) and diabetes (10.5%) were the most common co-morbidities among the participants.

**Table 1:** **Descriptive characteristics of studied population in a rural Vietnam**

Characteristics	N	(%)
Mean age (SD) 65.8 (9.9)		
Sex		
Men	112	40.7
Women	163	59.3
Education		
Under secondary school	165	60.0
Higher	110	40.0
Working status		
Currently working	101	36.7
Not working	174	63.3
Marital status		
Married	193	70.2
Single/widowed/divorced/separated	82	29.8
Religion		
None religion	116	42.2
Buddhist	126	45.8
Others	33	12.0
Economic status		
Normal households	262	95,3
Poor households	13	4,7
Physical activities		
Low level	116	42.2
Moderate level	145	52.7
High level	14	5.1
Duration of illness		
<1 year	73	26.5
1 to less than 5 years	147	53.5
5 to less than 10 years	29	10.5
10+ years	26	9.5
Adherence to treatment		
No	128	46.5
Yes	147	53.5
Presence of co-morbidity		
Diabetes	29	10.5
Kidney diseases	6	2.2
Cardiac diseases	42	15.3
Stroke	4	1.5
Arthritis	39	14.2

### Measurement properties of the WHOQOL-BREF

The results demonstrate good internal consistency of the WHOQOL-BREF among the hypertensive patients. The Cronbach’s alpha coefficient of WHOQOL-BREF for all 26 items was adequate (0.886). Across domains, the Cronbach’s alpha ranged from good to excellent, ranging at 0.796 for physical health domain, 0.683 for psychological health domain, 0.829 for social relationship domain and 0.654 for environmental health domain.

The validity of domain structure was assessed using Pearson’s Correlation Coefficients (Table 2). Statistically significant correlations were found among two overall items; and all domains, with most correlations greater than 0.4.

**Table 2:** **Correlation coefficients in two overall questions and four domains of WHOQOL**-**BREF**

	Q1	Q2	DOM1	DOM2	DOM3	DOM4
**Q1**	1					
**Q2**						
Correlation coefficient	0.6116	1				
p-value	<0.001					
Physical health (DOM1)						
Correlation coefficient	0.4583	0.472	1			
p-value	<0.001	<0.001				
Psychological health (DOM2)						
Correlation coefficient	0.4712	0.5048	0.6535	1		
p-value	<0.001	<0.001	<0.001			
Social relationships (DOM3)						
Correlation coefficient	0.2812	0.3014	0.3385	0.4615	1	
p-value	<0.001	<0.001	<0.001	<0.001		
Environment (DOM4)						
Correlation coefficient	0.4647	0.3706	0.5648	0.677	0.4582	1
p-value	<0.001	<0.001	<0.001	<0.001	<0.001	

### Quality of life profile and its associated factors among hypertensive patients

Table 3 presents WHOQOL-BREF average score of different domains. The highest average score of satisfaction were found in the Social relationship domain (64.1, (SD 14.1)), while the lowest average score were found in the Psychological domain (49.4, (SD 12.7)).

**Table 3:** **Quality of life domain scores** (**N** = **275**)

QOL domains	Min	Max	Mean (SD)
Physical (DOM1)	19	88	54.7 (14.9)
Psychological (DOM2)	13	81	49.4 (12.7)
Social relationships (DOM3)	19	100	64.1 (14.1)
Environment (DOM4)	25	88	59.5 (10.4)

The mean score of each domain across socioeconomic characteristics is presented in Table 4. The mean of all four domains was significantly higher in those with the following characteristics: men, higher education, married, moderate level of physical activities, and adherence to treatment. There were significant differences across strata of working status, duration of illness, and present of co-morbidity in mean score of the four domains.

**Table 4:** **Bivariate associations between independent variables and quality of life**

Characteristics	QOL scores			
	Physical health (Mean (SD))	Psychological health (Mean (SD))	Social relationships (Mean (SD))	Environment (Mean (SD))
Age				
Coefficient	- 0.49	- 0.34	- 0.09	- 0.19
p	< 0.001	< 0.001	0.29	0.002
Sex				
Men	57.6 (13.7)	52.7 (11.9)	66.3 (12.2)	62.1 (9.9)
Women	52.7 (15.5)	47.2 (12.9)	62.6 (15.1)	57.7 (10.3)
p	0.006	< 0.001	0.026	< 0.001
Education				
Under secondary school	52.8 (15.3)	47.8 (12.4)	62.6 (14.3)	57.9 (9.9)
Higher	57.6 (13.9)	51.8 (12.9)	66.3 (13.4)	61.9 (10.7)
p	0.01	0.012	0.035	0.002
Working status				
Currently working	59.8 (13.5)	51.7 (11.9)	65.9 (13.2)	61.7 (9.8)
Not working	51.8 (14.9)	48.1 (13.0)	63.0 (14.5)	58.3 (10.5)
p	< 0.001	0.026	0.094	0.008
Marital status				
Married	57.4 (13.8)	51.8 (11.6)	66.4 (12.6)	61.4 (9.5)
Single/widowed/divorced/separated	48.4 (15.6)	43.8 (13.6)	58.8 (15.9)	55.1 (11.1)
p	< 0.001	< 0.001	< 0.001	< 0.001
Religion				
Buddhist	55.1 (15.4)	49.3 (12.5)	63.1 (14.9)	58.8 (11.1)
None religion	54.1 (13.9)	49.7 (12.9)	65.5 (12.7)	60.5 (9.8)
Others	55.6 (16.9)	49.1 (13.2)	63.1 (14.8)	58.7 (9.7)
p	0.845	0.961	0.357	0.417
Economic status				
Normal households	54.9 (14.5)	49.7 (12.4)	64.3 (13.9)	59.8 (10.1)
Poor households	51.1 (22.5)	43.8 (17.9)	60.1 (16.0)	54.1 (13.9)
p	0.554	0.262	0.299	0.053
Physical activities				
Low level	47.9 (13.3)	43.9 (10.8)	60.6 (15.6)	56.0 (9.9)
Moderate level	60.1 (13.9)	53.8 (12.4)	67.3 (11,82)	62.4 (9,7)
High level	55.5 (15.8)	50.1 (13.7)	60.4 (15.6)	58.6 (12.2)
p	< 0.001	< 0.001	0.004	< 0.001
Duration of illness				
<1 year	59.5 (14.3)	52.3 (11.8)	63.4 (15.8)	59.9 (9.8)
1 to less than 5 years	54.7 (14.8)	48.9 (13.0)	65.3 (12.6)	59.7 (10.4)
5 to less than 10 years	51.6 (10.8)	50.3 (9.4)	65.3 (9.6)	62.0 (8.6)
10+ years	45.0 (16.0)	42.8 (14.4)	58.2 (19.0)	54.9 (12.9)
p	< 0.001	0.01	0.548*	0.079
Adherence to treatment				
No	48.0 (13.4)	44.2 (11.1)	61.2 (14.7)	56.1 (10.4)
Yes	60.6 (13.7)	53.9 (12.4)	66.6 (13.0)	62.5 (9.4)
p	<0.001	<0.001	0.002	<0.001
Presence of co-morbidity				
No	58.5 (13.7)	50.5 (12.5)	64.5 (13.5)	60.4 (10.2)
Yes	48.7 (14.8)	47.6 (12.9)	63.4 (14.9)	58.0 (10.4)
p	<0.001	0.069	0.507	0.056

The results of multivariate linear regression are presented in Table 5. After adjusting for the other covariates in the model marital status and physical activities level were the only factors statistically significantly associated with all four domains of QOL. Adherence to treatment was associated with physical health, psychological health and environmental health domain. Age of participants was only associated with physical health and psychological health domain. Sex was only associated with psychological health. Similarly, education level was only associated with environmental health and presence of co-morbidity only associated with physical health. Overall, the multivariate analysis showed being male, married, attainment of higher education, having physical activity at a moderate level, and adherence to treatment were positively associated with QOL. However, older age and presence of co-morbidity were negatively associated with QOL.

**Table 5:** **Backward multiple linear regression analyses of significant factors associated with QOL among people living with hypertension**

	Physical health		Psychological health		Social relationships		Environment	
	Coef ^+^	95% CI	Coef	95% CI	Coef	95% CI	Coef	95% CI
**Age**	-0.32***	-0.47, -0.17	-0.21**	-0.35, -0.07				
**Sex**								
Men vs. women			3.46*	0.52, 6.39				
**Education**								
Higher vs. Under secondary school							1.56*	0.34, 2.78
**Marital status**								
Married vs. single/widowed	4.51**	1.22, 7.81	3.45*	0.18, 6.72	6.83***	3.31, 10.36	4.63***	2.10, 7.15
**Physical activities** (vs. none/low level)								
Moderate level	5.32**	1.86, 8.79	5.21**	2.05, 8.38	5.62**	2.29, 8.94	3.36*	0.67, 6.04
High level	-0.60	-7.51, 8.79	0.63	-5.73, 6.98	-1.95	-9.46, 5.54	0.39	-4.92, 5.71
**Adherence to treatment**								
Yes vs. No	8.12***	4.80, 11.44	6.14***	3.11, 9.17			4.17**	1.58, 6.77
**Presence of co**-**morbidity**								
Yes vs. No	-7.63***	-10.61, -4.65						

*p < 0.05; **p < 0.01; ***p < 0.001; + coefficient.

## Discussion

To our knowledge, this is one of first studies in Vietnam using WHOQOL-BREF measuring QOL among hypertensive patients living in the rural area. The highest mean satisfaction rating found in the social relationship domain reflected good feelings in personal relationships; fairly good sharing/support from family and friends; and good fulfillment from sexual activity. Conversely the lowest score found in psychological health indicated less positive but more negative feelings about life; not being good about ability of thinking, learning, memory and concentration; and poor self-esteem. The study among hypertensive people in Brazil found comparable results with the highest mean satisfaction for social relationship [28]. However, the study in Brazil found a higher range in score of QOL from 59.7 to 72.3.

In ours study analysis of how the domain scores varied according to socio-demographic characteristics yielded some interesting results. A decrease in QOL was observed with age, but only in relation to physical and psychological health rather than social relationship and environment health. Our result is similar to result of another study in a rural North of Vietnam [14]. However, that study measured changes in QOL by age in older people irrespective of specific disease group. A similar result was also found in another study conducted among hypertensive patients in Poland [29].

In line with others studies conducted in Vietnam [12, 14, 30] and elsewhere [21, 29], we found a variation in QOL by gender. However, although other studies in Vietnam have showed evidence of gender disparity in QOL, they have not captured the difference across domains of QOL. In our study, gender gap was only statistically significant in the psychological domain although a higher mean score of satisfaction was observed in men for all domains of QOL.

Education has been widely identified as a determinant of QOL; people with higher levels of educational often report better QOL [21, 31]. In our study, those attaining high school or higher education had significantly higher mean scores in the environment domain. This suggests the effect of education on a sense of safety and security of hypertensive patients.

Our study found a significantly higher QOL across all four domains among married participants compared with those who were widowed/separated/single. This supports the previous finding of a qualitative study conducted in the northern part of Vietnam [13], which suggested that married life creates a sense of completeness and contentedness. This result is also in line with that of previous studies in Vietnam [30], Malawi [27], and Indonesia [31].

It is interesting to note the positive relationship between physical activity (especially moderate level) and QOL in all four domains (physical, psychological, social and environmental after controlling other significant variables). It may be possible that the relationship is mediated by the impact of physical activity on controlling blood pressure, which leads to better QOL.

The association between adherence to treatment and QOL among hypertensive patients has been examined in literature [32–35]. These studies provide inconsistent results ranging from weak to strong correlation between QOL and adherence to treatment. Possible reasons for the disparate results are a difference in (i) the instrument used to measure QOL (WHOQOL-BREF vs. EQ-5D or SF36 or SF-12) and (ii) the definition of treatment compliance. In our study, the results revealed a strong correlation of adherence to treatment with QOL in physical, psychological health and environment.

A negative association between the presence of co-morbidity and QOL in physical health was suggested by our result. This result is consistent with that of other studies conducted in hypertensive patients in Poland [29] and China [36], although these studies used different instruments (SF-12 and SF-36) to measure QOL. Similarly, a review of QOL using SF-36 among hypertensive patients indicated that individuals with co-existent chronic conditions tend to have lower QOL, especially for physical health [10].

The results of our study have confirmed that WHOQOL-BREF is a reliable instrument to measure QOL among hypertensive patients. This is important finding for those wishing to use WHOQOL-BREF in future studies in Vietnam. The lower QOL among hypertensive patients with co-morbidities supports the importance of early diagnosis and effective treatment of chronic conditions to preserve QOL in these patients. Hypertensive patients with medium level of physical activity showed better QOL compared with their counterparts. Facilitating physical activity in the community, especially for hypertensive patients should be encouraged to improve QOL. Further study should investigate interventions for improving and preserving QOL of hypertensive patients especially for vulnerable groups including older age, women, unmarried patients and person with lower education.

Our study has some strengths and limitations needed to consider when interpreting the results. The strength of the study is that it employed WHOQOL-BREF to measure quality of life. The instrument has been proved having acceptable reliability and validity in a cross-sectional study across 23 countries [19]. This is one of few studies in QOL from Vietnam, and the first reporting from Southern Vietnam. Using random sampling, our sample was reflective of the demographics of the hypertensive population in the rural area. Further the demographics in the population are highly reflective of other communities in the developing countries and are thus of use outside the immediate population. This proposition is strengthen by the finding that our results are similar to other studies in developing countries [37, 38]. However, this is a cross-sectional study therefore it is limited, to assessing the association, rather than causality between QOL and other factors. The sample size was calculated to be appropriate for estimating the mean of QOL, and thus, may be underpowered with respect to detection of the association of some factors with QOL due to small subgroup sample sizes. This may have resulted in an inability to detect associations that were really present (Type II error), making our results conservative. Nevertheless, the study has observed statistical significance in many variables; this indicated the study had enough power in those. The participants were restricted at age 50+ years and in a rural area. Thus, caution should be taken when generalising the results outside this age demographic. The study was not designed to fully validate the Vietnamese version of WHOQOL-BREF and thus analysis of this was not exhaustive.

## Conclusion

This study has shown moderate QOL among hypertensive patients living in a rural area in southern part of Vietnam crossing all domains, except for psychological health, which was fairly low. Physical activities and marital status were important independent factors affecting all domains in QOL. Older age was associated with lower QOL in both physical and psychological health. Women with hypertension had lower satisfaction rating in psychological health than men. Presence of co-morbidity in hypertension patients is an important health issue influencing their satisfaction in physical health. Interventions targeted towards improving QOL of disadvantage patients are needed in the setting.
